# Mixed alcoholic fermentation of *Schizosaccharomyces pombe* and *Lachancea thermotolerans* and its influence on mannose-containing polysaccharides wine Composition

**DOI:** 10.1186/s13568-019-0738-0

**Published:** 2019-02-02

**Authors:** Ángel Benito, Fernando Calderón, Santiago Benito

**Affiliations:** Department of Chemistry and Food Technology, Polytechnic University of Madrid, Ciudad Universitaria S/N, 28040 Madrid, Spain

**Keywords:** *Schizosaccharomyces*, Galactomanoprotein, *Lachancea*, Mannoprotein, Wine, Polysaccharides

## Abstract

This study researched the winemaking performance of new biotechnology involving the cooperation of *Lachancea* and *Schizosaccharomyces* genera in the production of wine. In all fermentations where *Lachancea thermotolerans* was involved, higher lactic acid concentrations appeared, while all fermentations where *Schizosaccharomyces pombe* was involved, lower levels in malic acid concentration took place. The sensorial properties of the final wines varied accordingly. Differences in mouthfeel properties and acidity occurred in the different fermentation trials. Fermentations with the highest concentration of hydrolyzed mannose showed the highest mouthfeel properties, but the lack of acidity reduced their overall impression. Wines made from a combination of *L. thermotolerans* and *S. pombe* showed the highest overall impression and were preferred by the tasters due to the balance between mouthfeel properties and acidity.

## Introduction

Several studies have proven that specific non-*Saccharomyces* strains are able to improve wine quality (Fleet 2008; Jolly et al. 2014; Varela 2016; Padilla et al. 2016), resulting in the use of these non-*Saccharomyces* yeast species in winemaking. During the past years, alternatives to conventional alcoholic fermentation and malolactic fermentation performed by *Saccharomyces cerevisiae* and *Oenococcus oeni* have become available to avoid specific collateral effects such as high concentrations of acetic acid or biogenic amines, which take place under specific conditions such as those that occur in warm viticulture areas (Benito et al. 2015a). Combined fermentation (co-inoculation) involving *Lachancea thermotolerans* (formerly known as *Kluyveromyces thermotolerans*) and *Schizosaccharomyces pombe* seems to be the appropriate for warm viticulture areas such as Spain (Benito et al. 2016a; Benito 2018).

The deacidification ability of *S. pombe* allows the conversion of harsh-tasting l-malic acid to ethanol (Benito et al. 2016c) which result in acidic grape juice from northern Atlantic European grape growing regions to become smoother. However, several collateral effects described for *S. pombe*, such as the production of high concentrations of acetic acid are common when this species is used in winemaking (Benito et al. 2014; Roca-Domènech et al. 2018) or other fermentation industries (Minnaar et al. 2017; Satora et al. 2018). Fleet (2008) proposed that through proper programs of yeast selection, specific strains could perform fermentation processes without the formation of excessive acetic acid, ethyl acetate, hydrogen sulphide and sulphur dioxide, or other off-flavors. For example, recent research reported fermentations with low acetic acid concentrations (Benito et al. 2014; Domizio et al. 2017; Du Plessis et al. 2017) that varied from 0.1 to 0.34 g/L, while other authors reported values above 1 g/L (Mylona et al. 2016; Miljic et al. 2017) depending on the strain used.

Other *Schizosaccharomyces* uses besides conventional malic acid deacidification have been reported during the last few years (Benito et al. 2018). *Schizosaccharomyces* can reduce gluconic acid concentrations (Peinado et al. 2009) and improve wine quality made from spoiled grape juice. It also improves wine color through the production of stable pigments such as vitisins (Benito et al. 2017). It can also avoid the formation of biogenic amines and ethyl carbamate concentrations to produce healthier wines from a food safety point of view (Mylona et al. 2016). Another advantage is the polysaccharide release during aging over lees or fermentation (Palomero et al. 2009; Domizio et al. 2017), which improves mouth sensory properties.

*Lachancea thermotolerans* is able to increase the acidity in low acidic musts from Mediterranean warm regions (Kapsopoulou et al. 2005, 2007; Gobbi et al. 2013; Balikci et al. 2016; Benito et al. 2016b; Domizio et al. 2016) through the production of lactic acid, thereby improving sensory properties. The use of *Lachancea thermotolerans* has recently become popular in modern enology because of the advantages such as biocontrol applications that inhibit the presence of spoilage microorganisms (Nally et al. 2018; Benito 2018). Even though there is only one commercial strain available, some researchers are performing selection processes in order to increase the number of available clones (Escribano et al. 2018).

Mannoproteins are the second most abundant family of polysaccharides after arabinogalactan-proteins that originate from grapes (Vidal et al. 2003). Mannoproteins are released into wine from yeast cell walls during fermentation and ageing over lees (Palomero et al. 2009; Domizio et al. 2017). Previous studies have demonstrated a positive effect of polysaccharides on the quality and sensorial properties of wine (Vidal et al. 2004; Gawel et al. 2014). Polysaccharides affect mouth-feel properties such as fullness, while reducing the astringency of the final product (Vidal et al. 2004) and contribute to the retention of positive aroma compounds (Lubbers et al. 1994).

The technology based on the use of *Lachancea* and *Schizosaccharomyces* genera was studied before for simple fermentation parameters (Benito et al. 2015a). During the last year, more advanced parameters such as volatile compounds, amino acids, biogenic amines (Benito et al. 2016b) and anthocyanin composition (Benito et al. 2017) have also been studied. However, several additional fermentation factors require to be researched for this modern technology, and to this end, our research focus on the effect of *Lachancea* and *Schizosaccharomyces* genera on wine mannose-containing polysaccharides release during alcoholic fermentation.

## Materials and methods

### Microbiological material

The yeast strains selected for the trials were: *Kluyveromyces thermotolerans* Concerto™ (Hansen, Hørsholm, Denmark), *S. cerevisiae* CECT 87 (Type Culture Collection of Spain, Valencia University, Spain) and a pre-commercial *S. pombe* V2 [GenBank accession number HE963293; also deposited and publicly available in the Chemistry and Food Technology Department Yeast Collection of Polytechnic University of Madrid; Benito et al. (2014)]. The selected lactic acid bacteria strain was *O. oeni* 217 (Type Culture Collection of Spain, Valencia University, Spain).

### Vinification

The experimental vinifications took place at a scale according to previously described microvinification methodology (Sampaio et al. 2007), which was modified (Belda et al. 2015; Benito et al. 2015a, b). Tempranillo grape must (Rioja Alta, Spain) was used with 226 g/L sugar, pH = 3.61, PAN 333 mg/L, malic acid was 2.54 g/L and citric acid was 030 g/L. Lactic and acetic acids were 0.01 g/L.

Fermentations took place in 5 L vessels where 4 L of must fermented in triplicate for each treatment. The free run Tempranillo must after being destemmed and crushed was autoclaved at 105 °C for 5 min. The initial inoculum concentration for the different treatments were *S. cerevisiae* alone (10^6^ cfu/mL) (SC), *L. thermotolerans* (10^6^ cfu/mL) and *S. cerevisiae* (10^6^ cfu/mL) 72 h later (LT…SC), *L. thermotolerans* (10^6^ cfu/mL) and *S. pombe* (10^6^ cfu/mL) 72 h later (LT…SK) and *S. pombe* alone (10^6^ cfu/mL) (SK). The alcoholic fermentations took place at controlled temperature of 25 °C. Fermentations regarding *S. cerevisiae* alone (SC) were inoculated with *O. oeni* (10^7^ cfu/mL) and performed malolactic fermentation in 2.8 L vessels at 18 °C. Once fermentations were over the wines were racked into small vessels of 2.8 L where they settled at 4 °C for 7 days. After that period, the supernatant was introduced into 750 mL bottles where 100 mg/L of potassium metabisulfite (Agrovin S.A, Alcazar de San Juan, Spain) were added. The bottles were sealed and stayed horizontally in a refrigerator at 4 °C. The sensory session took place 58 days after the last fermentation ended.

### Biochemical compounds

The quantification of parameters showed in Table 1 were performed using the method described in previous studies (Belda et al. 2015; Benito et al. 2015b). A Y15 Autoanalyser (Biosystems, Barcelona, Spain), a GAB Microebu and a Crison pH meter (Basic 20, Crison Barcelona, Spain) were used.

**Table 1:** Final analysis of fermentations from original must of Tempranillo grapes: *S. cerevisiae* 87 alone (SC), sequential fermentation with *S. cerevisiae* 87 and *L. thermotolerans* CONCERTO™ (LT…SC), sequential fermentation with *Schizosaccharomyces pombe* V2 and *L. thermotolerans* CONCERTO™ (LT…SK), *Schizosaccharomyces pombe* 4.5 alone (SK), and fermentations after a malolactic fermentation with *Oenococcus oeni* 217 (+MLF)

Compounds	SC	SC + MLF	LT…SC	LT…SC + MLF	LT…SK	SK
l-Lactic acid (g/L)	0.01 ± 0.01 a	1.46 ± 0.05 b	1.63 ± 0.14 c	3.11 ± 0.21 e	1.86 ± 0.19 d	0.01 ± 0.01 a
l-Malic acid (g/L)	2.43 ± 0.03 b	0.01 ± 0.01 a	2.39 ± 0.05 b	0.01 ± 0.01 a	0.01 ± 0.01 a	0.01 ± 0.01 a
Acetic acid (g/L)	0.28 ± 0.01 a	0.39 ± 0.02 b	0.25 ± 0.03 a	0.34 ± 0.04 b	0.30 ± 0.04 ab	0.36 ± 0.02 b
Glucose + fructose (g/L)	1.55 ± 0.19 b	0.07 ± 0.03 a	1.61 ± 0.24 b	0.05 ± 0.02 a	1.72 ± 0.25 b	1.58 ± 0.16 b
Glycerol (g/L)	7.12 ± 0.02 a	7.17 ± 0.05 a	7.14 ± 0.06 a	7.19 ± 0.11 ab	7.39 ± 0.09 b	7.78 ± 0.03 c
pH	3.64 ± 0.02 b	3.73 ± 0.02 c	3.47 ± 0.03 a	3.58 ± 0.06 b	3.53 ± 0.05 ab	3.91 ± 0.02 d
Urea (mg/L)	1.78 ± 0.06 b	1.97 ± 0.08 c	1.82 ± 0.09 bc	2.11 ± 0.11 d	0.06 ± 0.03 a	0.03 ± 0.01 a
Citric acid (g/L)	0.29 ± 0.01 b	0.02 ± 0.01 a	0.27 ± 0.02 b	0.04 ± 0.02 a	0.29 ± 0.03 b	0.27 ± 0.02 b
Ethanol (% *v*/*v*)	13.78 ± 0.02 c	13.80 ± 0.05 c	13.72 ± 0.06 cb	13.70 ± 0.09 cb	13.62 ± 0.05 b	13.55 ± 0.04 a
Acetaldehyde (mg/L)	34.16 ± 1.55 c	1.88 ± 0.33 a	29.55 ± 2.13 b	1.79 ± 0.24 a	46.38 ± 2.96 d	58.36 ± 2.55 e
Pyruvic acid (mg/L)	58.56 ± 3.55 b	13.67 ± 3.79 a	62.42 ± 5.73 b	17.82 ± 6.21 a	122.63 ± 9.15 c	168.82 ± 5.78 d

Results are the mean ± SD of three replicates. Means in the same row with the same letter are not significantly different (*p* < 0.05)

### Yeast growth

**Fig. 1:**
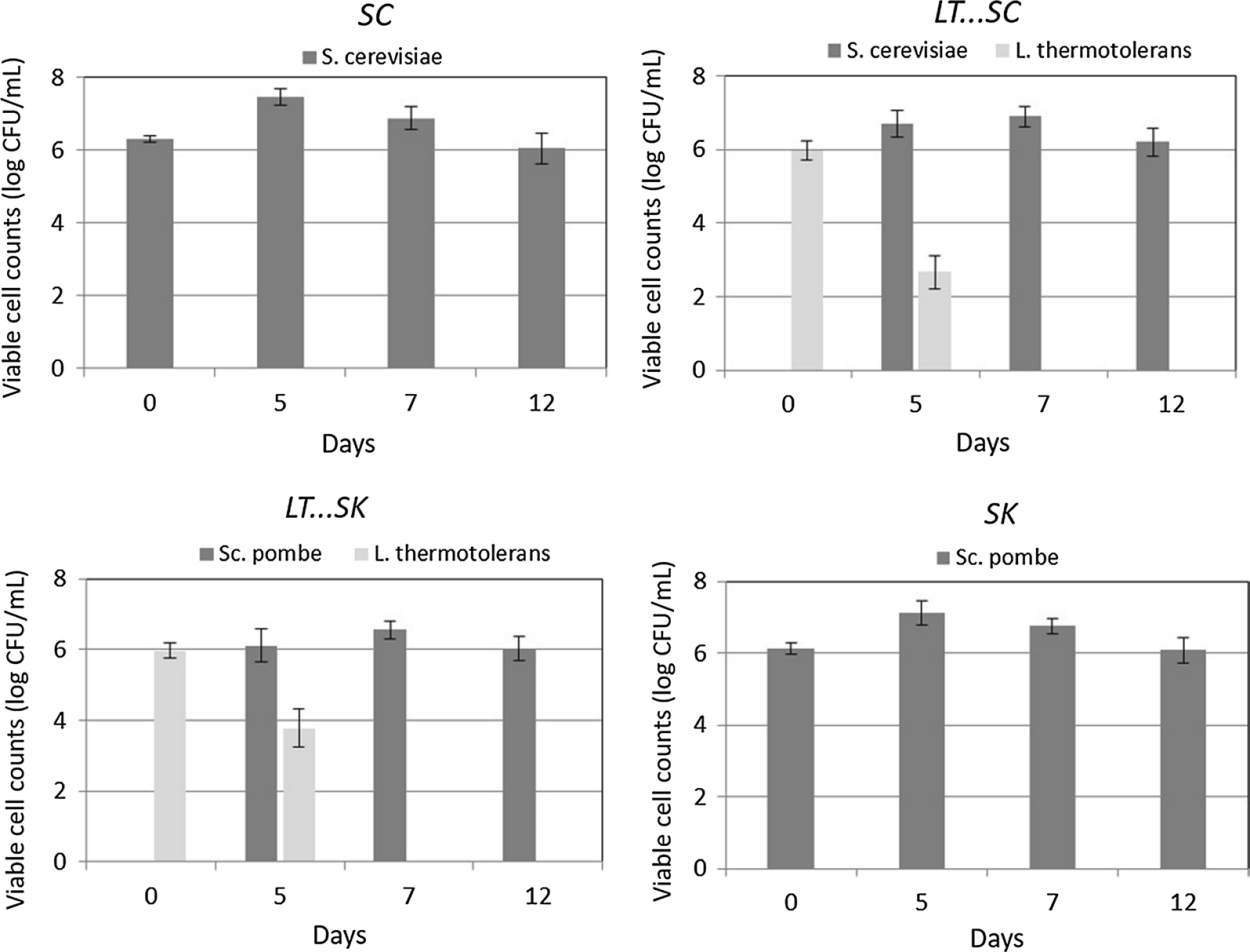
Change in the population of *S. cerevisiae* 87 alone (SC), sequential fermentation with *Saccharomyces cerevisiae* 87 and *L. thermotolerans* CONCERTO™ (LT…SC), sequential fermentation with *S. pombe* V2 and *Lachancea thermotolerans* CONCERTO™ (LT…SK) and *S. pombe* V2 alone (SK). Values are means ± standard (logCFU/mL) deviations for three independent fermentations

The changes in the population of the different yeast species (Fig. 1) were studied according to the methodology described by Benito et al. (2015b), which is based on the use of selective-differential media such as YEPDAactBzCL *Schizosaccharomyces* selective media (Benito et al. 2018), lysine media (Morris and Eddy 1957), YEPD media (Kurtzman et al. 2011) and MRS agar (Oxoid, Basingstoke, UK). *Schizosaccharomyces* selective media allows monitoring *Schizosaccharomyces* colonies, lysine media allows to detect some non-*Saccharomyces* yeasts such as *L. thermotolerans*, YEPD media alows to detect any wine yeast species and MRS agar allows monitoring lactic bacteria.

### Determination of mannose

Mannose content of the total soluble wine polysaccharides was evaluated according to the methodology described by Belda et al. (2016).

### Sensory analysis

The final wines were assessed in a blind tasting by a panel of 15 experienced wine tasters, all staff members of the Chemistry and Food Technology Department of Polytechnic University of Madrid (Madrid, Spain) and the Accredited Laboratory Estación Enológica de Haro (Haro, Spain). The sensory analysis was similar to that described in previous works (Belda et al. 2015, 2016; Benito et al. 2017). In this study, 14 attributes were established by consensus (Fig. 3).

### Statistical analysis

All statistical analyses were performed using PC Statgraphics v.5 software (Graphics Software Systems, Rockville, MD, USA). The significance was set to *p* < 0.05 for the ANOVA matrix *F* value. A multiple range test was used to compare the means.

## Results

### Fermentation performance

Figure 1 shows the yeast counts during the different fermentations. *S. cerevisiae* and *S. pombe* cells remained constant until the conclusion of fermentation in concentrations that varied from 8.4 × 10^5^ to 6.1 × 10^6^ cfu/mL. *L. thermotolerans* cell counts decreased after day 5.

*Schizosaccharomyces pombe* degraded all malic acid (Table 1) during alcoholic fermentation (AF) in pure and mixed modalities, while *S. cerevisiae* degraded malic acid only to about 5% (Table 1). *O. oeni* converted the remaining malic acid into lactic acid to obtain stable wines in trials fermented by *S. cerevisiae* (Table 1). *L. thermotolerans* synthetized l-lactic acid during AF (Table 1). The final l-lactic acid concentrations varied from 1.46 g/L for the case fermented by *S. cerevisiae* and *O. oeni*, to 3.11 g/L for the case fermented by *L. thermotolerans*, *S. cerevisiae* and *O. oeni*. The final pH varied from 3.47 to 3.91 g/L due to malic and lactic acid metabolism. Wines produced with *S. pombe* had a pyruvic acid concentrations of 168 mg/L and a glycerol concentration of about 7.78 g/L. The reported acetic acid concentrations were below 0.4 g/L. Ethanol concentrations varied from 13.55 to 13.80% (v/v). Wines produced with *S. pombe* had slightly lower ethanol concentration of 0.23% (v/v) than *S. cerevisiae* (control) wines. Fermentations involving *S. pombe* resulted in final urea concentrations lower than 0.1 mg/L (Table 1). The fermentations, which did not involve *S. pombe*, showed final urea concentrations of about 2 mg/L. Urea concentration increased from 0.2 to 0.3 g/L after malolactic fermentation (MLF). Malolactic fermentations performed by *O. oeni* showed final citric acid concentrations of 0.04 mg/L and below (Table 1). Slightly higher acetic acid concentrations were found in wines that underwent MLF. Those increases varied from 0.09 to 0.11 g/L.

**Fig. 2:**
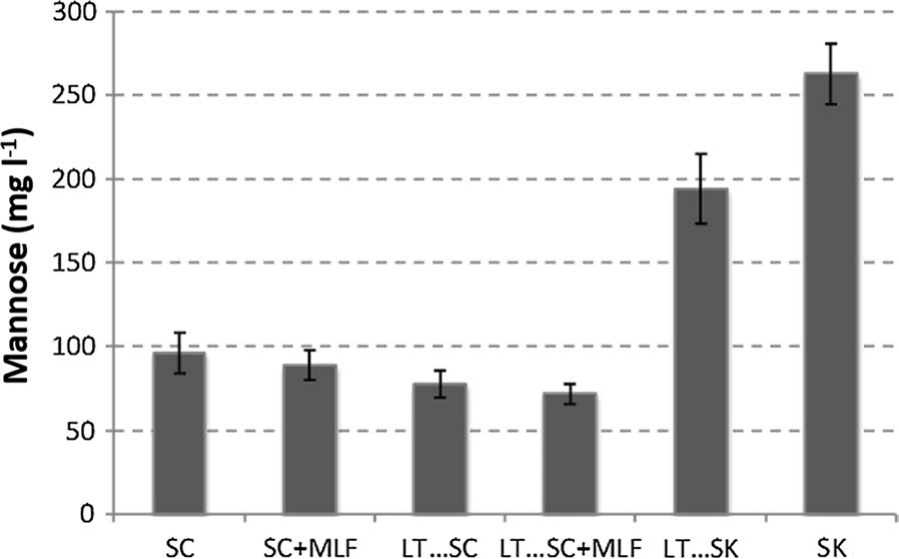
Mannose released from polysaccharides after hydrolysis of wines fermented at microvinification scale with: *S. cerevisiae* 87 alone (SC), sequential fermentation with *Saccharomyces cerevisiae* 87 and *L. thermotolerans* CONCERTO™ (LT…SC), sequential fermentation with *S. pombe* V2 and *Lachancea thermotolerans* CONCERTO™ (LT…SK), *S. pombe* V2 alone (SK), and fermentations after malolactic fermentation with *Oenococcus oeni* 217 (+MLF)

Figure 2 shows the content of mannose after the fermentations and cold sedimentation. *S. cerevisiae* and *L. thermotolerans* release mannose from mannoproteins while *S. pombe* release it from galactomannoproteins. The highest increase in mannose took place in the fermentations where *S. pombe* fermented alone. The final concentration was about 250 mg/L of mannose released from polysaccharides after hydrolysis. The sequential fermentations involving *L. thermotolerans* and *S. pombe* showed a significant increase in mannose, compared to *S. cerevisiae* fermentations only. The final difference was about 100 mg/L in mannose.

### Sensory evaluation

**Fig. 3:**
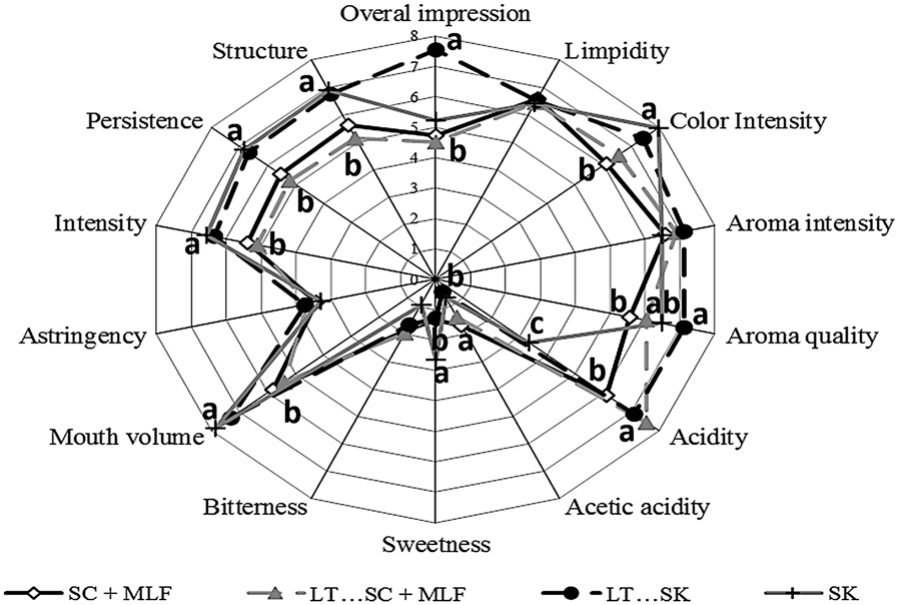
Results of the sensory analysis of bottled wines from different fermentation processes of *Saccharomyces cerevisiae* 87 alone (SC), sequential fermentation with *Saccharomyces cerevisiae* 87 and *L. thermotolerans* CONCERTO™ (LT…SC), sequential fermentation with *S. pombe* V2 and *L. thermotolerans* CONCERTO™ (LT…SK), *S. pombe* V2 alone (SK), and fermentations after malolactic fermentation with *Oenococcus oeni* 217 (+MLF)

Figure 3 shows a radar graph of the scores of various attributes. It shows differences in the perception of acidity, as several microorganisms are able to affect acidity. Color intensity was higher in wines produced without malolactic fermentation, compared to wines that underwent malolactic fermentation. None of the wines that were produced with *S. pombe* or *L. thermotolerans* showed any negative organoleptic properties. Significant differences in mouth volume, persistence, structure and aroma were evident between the different treatments (Fig. 3). Sequential fermentations by *Schizosaccharomyces* and *Lachancea* obtained the maximum mark in overall impression.

## Discussion

### Fermentation performance

Certain authors describe *L. thermotolerans* as a yeast not able to complete fermentation when the final alcohol levels are between 9 and 10% (Lubbers et al. 1994; Kapsopoulou et al. 2005; Benito et al. 2016b). For this reason, *L. thermotolerans* should be used in combination with yeast genera such as *Saccharomyces* or *Schizosaccharomyces* to complete the fermentation process (Benito et al. 2015a; Balikci et al. 2016). The combine AF finished the fermentations as the glucose/fructose concentrations were lower than 2 g/L (Table 1).

Other studies described L-lactic acid production of up to 6 g/L when *Lachancea* was utilized in pure fermentation (Gobbi et al. 2013; Benito et al. 2015b, 2016b). l-Lactic acid concentrations were higher in wines where grape juice was inoculated with *L. thermotolerans* where wines underwent MLF (*O. oeni*). This is a useful strategy to increase the acidity of wines from grapes originating from warm regions which usually have a low acidity (Benito et al. 2018).

The increase in acetic acid concentrations could be due to citric acid consumption of *O. oeni* during MLF. Other authors reported high levels of acetic acid as a possible collateral effect when MLF occurs without proper control (Mylona et al. 2016). The increases of about 0.1 g/L in acetic acid after MLF observed in this study support those theories (Table 1). Work by Comitini et al. (2011) and Gobbi et al. (2013) reported that *L. thermotolerans* produced lower concentrations of acetic acid than *S. cerevisiae* (Miljic et al. 2017) with differences varying between 0.18 and 0.33 g/L. In contrast to the above, the genus *Schizosaccharomyces* often produce acetic acid concentrations over 0.9 g/L (Mylona et al. 2016). Nevertheless, recent studies on *Schizosaccharomyces* showed that specific strains produce acetic acid only as low as 0.1 g/L (Domizio et al. 2017; Du Plessis et al. 2017; Roca-Domènech et al. 2018). Fleet (2008) proposed the selection of a *Schizosaccharomyces* strain to prevent conventional co-fermentation effects attributed to this genus, such as high acetic acid production. The final observed acetic acid concentration in the fermentations regarding *S. pombe* of about 0.35 g/L support the theories related to strain variability. The use of *S. pombe* under reduced osmotic stress conditions afforded by fed-batch alcoholic fermentation also allows the production of wines with low levels in acetic acid (Roca-Domènech et al. 2018).

Domizio et al. (2017) found concentrations of up to 430 mg/L pyruvic acid in wines made with *Schizosaccharomyces*. However, the pyruvic acid was measured 5 days after fermentation started when it reached maximum concentration during AF. Increased pyruvic acid formation is associated with increased concentrations of stable color pigments which can improve wine color (Benito et al. 2017; Benito 2018). In this study, the pure *S. pombe* fermentation produced 66% more pyruvic acid than the *S. cerevisiae* control. The mixed fermentation between *L. thermotolerans* and *S. pombe* showed a final pyruvic acid concentration of 50% higher than the *S. cerevisiae* control.

Non-*Saccharomyces* yeasts are one of the main contributors of glycerol content to wine quality (Jolly et al. 2006, 2014; Goold et al. 2017). Domizio et al. (2017) reported the production of glycerol of up to 11.4 g/L by certain strains of by *Schizosaccharomyces*. In this study the increases in glycerol produced by the non-*Saccharomyces* were moderated. In the case of *S. pombe* the increase was 0.66 g/L higher than the *S. cerevisiae* control, while in the case of the mixed fermentation between *L. thermotolerans* and *S. pombe* the increase was only of 0.27 g/L higher.

*Schizosaccharomyces* is tolerant to ethanol stress environments (Garcia et al. 2016). Other studies related to *L. thermotolerans* (Gobbi et al. 2013) and *S. pombe* (Benito et al. 2013) reported similar results. Although the ethanol concentration (Table 1) were significantly different, the differences were lower than 0.25% (v/v). Ethanol reduction higher than 1% (v/v) appear to be related to conditions of increased aeration (Contreras et al. 2015; Morales et al. 2015), or specific enzyme activity such as glucose oxidase or catalase (Rocker et al. 2016). These methodologies can be applied to avoid difficult fermentations of grape must with a high sugar concentration. In those cases, it is difficult for regular yeasts to convert all sugars into ethanol.

The enzymatic urease ability of *S. pombe* is valuable for producing wines free of ethyl carbamate (Mylona et al. 2016), which is important from a food safety point of view as ethyl carbamate is considered to be a carcinogenic hazard. In this study the fermentations where *S. pombe* was involved showed urea levels 97% lower than the controls. As urea is the main precursor of ethyl carbamate in wine (Benito et al. 2016c), the wines that showed final urea concentrations close to 0 mg/L look to be virtually stable against future ethyl carbamate production.

### Mannose-containing polysaccharides content in fermentations

The increase of mannoprotein concentrations during AF is a modern approach to improve wine quality (Domizio et al. 2014). Domizio et al. (2017) reported on the special ability of the *Schizosaccharomyces* genus to release high amounts of polysaccharides. *S. pombe* releases galactomannoproteins instead of mannoproteins (Domizio et al. 2017). Those galactomannoproteins contains a higher content in mannose ranging from 44 to 47% than galactose, that ranges from 36 to 45% for the case of *S. pombe* (Domizio et al. 2017). On the other hand, *L. thermotolerans* is a moderate mannoprotein producer when compared to *S. cerevisiae* (Belda et al. 2016). Non-*Saccharomyces* species such as *T. delbrueckii* also produce higher concentrations of mannoproteins when compared to *S. cerevisiae* during AF (Belda et al. 2015). The results of this study show higher release of mannose in all fermentations involving *S. pombe* (Fig. 2). This indicates a higher mannose-containing polysaccharides release during the alcoholic fermentation.

### Sensory evaluation

The high color intensity of wines of non-MLF is in agreement with previous work by Mylona et al. (2016) that found significant anthocyanin concentration decreases through lactic acid bacteria metabolism. *L. thermotolerans* metabolism improves wine color intensity due to the production of lactic acid thereby decreasing the pH (Benito 2018) while *S. pombe* producers high concentrations of vitisins or pyranoanthocyanins (Benito et al. 2017; Benito 2018). Mannoproteins can also increase the anthocyanins in wine (Vidal et al. 2003).

According to works by Vidal et al. (2004) and Gawel et al. (2014), factors related to mouth-feel properties such as fullness sensation or perceived viscosity, are dependent on polysaccharides concentrations. This is in agreement with mannose-containing polysaccharides levels reported in this study that showed high scores of mouth-feel properties such as structure or mouth volume (Table 1, Fig. 2).

Although the structure was similar in all fermentations involving *S. pombe*, sequential fermentations by *L. thermotolerans* and *S. pombe* obtained the maximum mark in overall impression due to a better balance between acidity and structure. Differences in aroma quality could be related to the ability of the mannoprotein to retain positive aroma compounds such as B-ionone (Lubbers et al. 1994) or the ability of *L. thermotolerans* to generate high levels of aromatic esters (Benito et al. 2015b).

The combination of *S. pombe* and *L. thermotolerans* as a new winemaking biotechnology is able to improve wine quality under specific conditions. It is a substitute to the conventional MLF, which increases mannose-containing polysaccharides of wine and maintain a balance between wine structure and acidity. The results of the fermentation trials showed positive differences in acetic acid, urea, pyruvic acid and glycerol concentrations as well as sensory attributes. The proposed inoculation combination could improve wine aging aptitude as it reduces the pH and prevents possible collateral effects such as the formation of biogenic amines or ethyl carbamate.

## Abbreviations

*S. cerevisiae* or SC: *Saccharomyces cerevisiae*
*S. pombe* or SK: *Schizosaccharomyces pombe*
*L. thermotolerans* or LT: *Lachancea thermotolerans*
